# Phenotypic and Genotypic Characteristics of Antimicrobial Resistance in *Citrobacter freundii* Isolated from Domestic Ducks (*Anas platyrhynchos domesticus*) in Bangladesh

**DOI:** 10.3390/antibiotics12040769

**Published:** 2023-04-17

**Authors:** Tarana Ahmed, Md. Saiful Islam, Najmul Haider, Linzy Elton, Badrul Hasan, Mohammad Nuruzzaman, Md. Tanvir Rahman, S. M. Lutful Kabir, Md. Shahidur Rahman Khan

**Affiliations:** 1 Department of Microbiology and Hygiene, Bangladesh Agricultural University, Mymensingh 2202, Bangladesh; dvm41257@bau.edu.bd (M.S.I.); tanvirahman@bau.edu.bd (M.T.R.); lkabir79@bau.edu.bd (S.M.L.K.); 2 Department of Livestock Services, Ministry of Fisheries & Livestock, Government of the Peoples Republic of Bangladesh, Krishi Khamar Sarak, Farmgate, Dhaka 1215, Bangladesh; 3 School of Life Sciences, Keele University, Staffordshire ST5 5BG, UK; n.haider@keele.ac.uk; 4 The Royal Veterinary College, University of London, Hertfordshire AL9 7TA, UK; 5 Centre for Clinical Microbiology, Department of Infection, Division of Infection and Immunity, Royal Free Campus, University College London, London NW3 2PF, UK; linzy.elton@ucl.ac.uk; 6 Department of Jobs, Precincts and Regions, AgriBio, Centre for AgriBioscience, Bundoora, VIC 3083, Australia; badrul.hasan@agriculture.vic.gov.au; 7 Ministry of Public Administration, Dhaka 1205, Bangladesh; mnzaman27@gmail.com

**Keywords:** *C. freundii*, antimicrobial resistance, multidrug resistance, resistance genes, ducks, public health, Bangladesh

## Abstract

Antimicrobial resistance (AMR) in *Citrobacter freundii* poses a serious challenge as this species is one of the sources of nosocomial infection and causes diarrheal infections in humans. Ducks could be the potential source of multidrug-resistant (MDR) *C. freundii*; however, AMR profiles in *C. freundii* from non-human sources in Bangladesh have remained elusive. This study aimed to detect *C. freundii* in domestic ducks (*Anas platyrhynchos domesticus*) in Bangladesh and to determine their phenotypic and genotypic antibiotic susceptibility patterns. A total of 150 cloacal swabs of diseased domestic ducks were screened using culturing, staining, biochemical, polymerase chain reaction (PCR), and matrix-assisted laser desorption/ionization time-of-flight (MALDI-TOF) to detect *C. freundii*. Phenotypic and genotypic antibiotic susceptibility patterns were done by the disk diffusion method and PCR, respectively. In total, 16.67% (25/150) of the samples were positive for *C. freundii*. *C. freundii* isolates showed a range of 20% to 96% resistance to cefotaxime, gentamicin, levofloxacin, ciprofloxacin, cotrimoxazole, tetracycline, ampicillin, and cephalexin. More than 60% of the isolates were phenotypically MDR, and the index of multiple antibiotic resistance ranged from 0.07 to 0.79. Genes encoding resistance to beta-lactams [*bla*_TEM-1_-88% (22/25), *bla*_CMY-2_-56% (14/25), *bla*_CMY-9_-8% (2/25), and *bla*_CTX-M-14_-20% (5/25)], sulfonamides [*sul1*-52% (13/25), *sul2*-24% (6/25)], tetracyclines [*tetA*-32% (8/25) and *tetB*-4% (1/25)], aminoglycosides [*aacC4*-16% (4/25)], and fluoroquinolones [*qnrA*-4% (1/25), *qnrB*-12% (3/25), and *qnrS*-4% (1/25)] were detected in the isolated *C. freundii*. To the best of our knowledge, this is the first study in Bangladesh to detect MDR *C. freundii* with their associated resistance genes from duck samples. We suggest addressing the burden of diseases in ducks and humans and associated AMR issues using the One Health approach.

## 1. Introduction

Antimicrobial resistance (AMR) is a major global issue that jeopardizes human, animal, and environmental health [1]. If nothing is done to curb AMR by 2050, it is expected to inflict hundreds of millions of fatalities worldwide, enormous financial consequences, and a significant decline in animal production [2,3]. *Citrobacter freundii* has become more resistant as a direct result of the widespread usage of antibiotics with a broad spectrum of activity [4]. Antimicrobials such as fluoroquinolones, aminoglycosides, nitrofurantoins, carbapenems, and cephalosporins are the typical classes of antibiotics used to treat infections caused by *C. freundii* [5]. However, the concern is rising because *C. freundii* has developed resistance to multiple antibiotics. Moreover, it is possible that low-virulent *Citrobacter* spp., which are able to survive in the host for a long time, could impact the evolution of pathogens by accumulating genes that code for resistance to multiple classes of antimicrobials [6]. The acquisition of resistance genes that confer resistance to multiple antibiotic classes from external sources, such as the environment or other bacteria, can cause multidrug resistance in *Citrobacter* species [7].

Antimicrobial-resistant bacteria may be present in duck droppings, contaminating the environment [8]. Ducks are possible carriers of important antimicrobial-resistant pathogens that might spread to humans because of their interactions with humans [9]. Humans can acquire *C. freundii* from ducks through contact with infected eggs, raw or undercooked meat, and duck carcasses at the slaughterhouse [10]. *C. freundii* infections can be fatal, with death rates ranging from 33 to 48% overall, including 30% mortality in children [11]. The central nervous system of survivor infants may be severely affected, resulting in extreme mental impairment, convulsions, and hemiparesis [12].

The poultry industry in Bangladesh has grown and become a successful agricultural business that makes a significant contribution to the country’s overall gross domestic product and provides a valuable source of protein [13,14]. Duck farming is a profitable livestock sector around the world because of the eggs, meat, and feathers it produces [15]. In Bangladesh, duck farming is important in its rural economy, second only to chicken production [16]. Ducks are typically reared in small-scale farming, either indoors or outdoors, or in an integrated farming system in Bangladesh [17], where they come or stay in close contact with humans. However, the greatest barrier to large-scale duck farming in Bangladesh is infectious disease outbreaks, including duck viral enteritis, duck viral hepatitis, avian influenza, botulism, duck cholera, etc. [18,19].

One of the bacterial pathogens found in duck droppings is *Citrobacter* spp. However, very little is known about the role of *Citrobacter* spp. as the source of infections in duck populations. *C. freundii* is the most prevalent among all *Citrobacter* species that causes infections in humans and animals [20]. *C. freundii* was isolated from young ducks having salpingitis [21]. The most common symptoms of *C. freundii* infection in ducks are discharge from nostrils, leg weakness, whitish diarrhea, recumbency, headshaking, and even sudden death [22].

In Bangladesh, the issue is compounded by the fact that poultry farmers come into direct contact with ducks during the rearing process, particularly when raising domestic ducks (*Anas platyrhynchos domesticus*) in their own homes. This direct interaction between humans and ducks increases the risk of transmission of *C. freundii* from domestic ducks to children, creating an even greater cause for concern. Human cases of *C. freundii* have been recorded in Bangladesh, India, and other Asian countries [23,24,25]; however, *C. freundii* cases in non-humans are not well described in these regions. In fact, this bacterium has not been well characterized in animals from any South Asian countries. The aim of this study was to detect *C. freundii* from cloacal swabs of ducks and determine their phenotypic and genotypic antibiotic resistance patterns to elucidate their potential negative impacts on human health.

## 2. Results

### 2.1. Occurrence of C. freundii Isolates

In the polymerase chain reaction (PCR) test, 25 of 150 samples were positive for *Citrobacter* spp. (16.67%, 95% CI: 11.55–23.45%) (Table 1). In MALDI-TOF analysis, all the *Citrobacter* spp. were detected as *C. freundii*. The occurrence of *C. freundii* in cloacal swabs of ducks was higher but not significant in the Kishoreganj district (22%, 95% CI: 12.75–35.24%) compared to that of Mymensingh (18%, 95% CI: 9.77–30.80%) and Netrokona (10%, 95% CI: 4.35–21.36%) districts (Table 1).

### 2.2. Phenotypic Antibiogram Profiles of Isolated C. freundii

**Figure 1:**
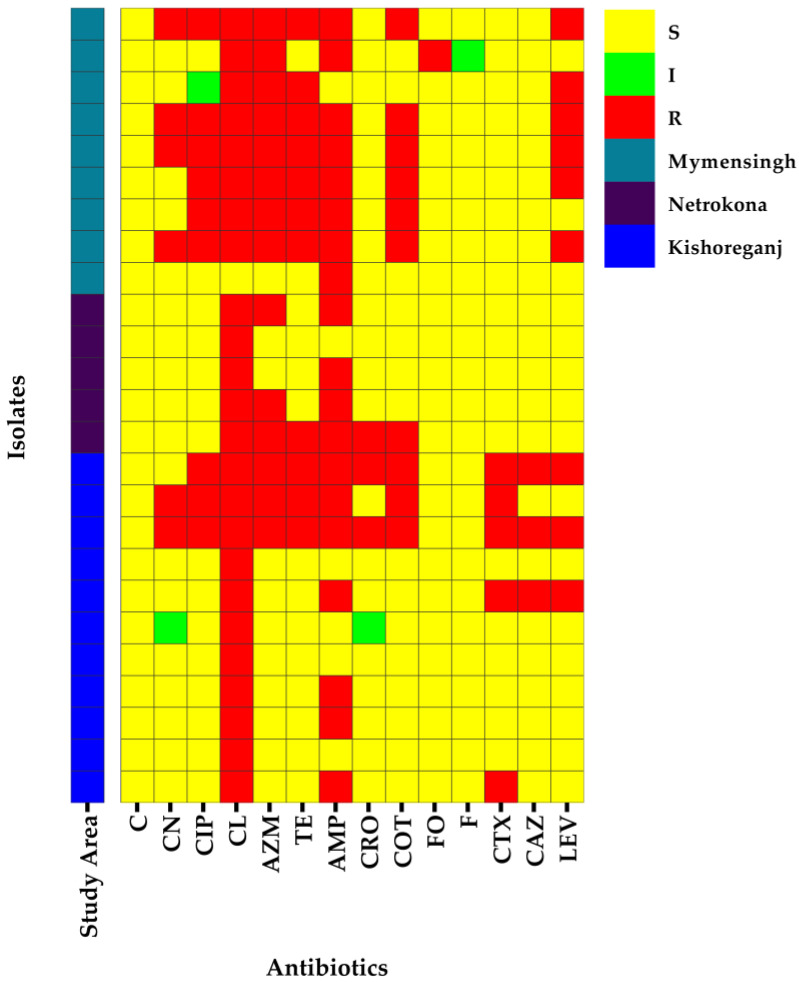
Heatmap showing the distribution of antibiogram profiles in 25 *C. freundii* isolates detected from cloacal swabs of ducks, LEV = levofloxacin; CAZ = ceftazidime; CTX = cefotaxime; F = nitrofurantoin; FO = fosfomycin; COT = cotrimoxazole; CRO = ceftriaxone; AMP = ampicillin; TE = tetracycline; AZM = azithromycin; CL = Cephalexin; CIP = ciprofloxacin; CN = gentamycin; C = chloramphenicol, S = Sensitive, I = Intermediate, R = Resistant.

In the antibiotic susceptibility test (AST), *C. freundii* isolates showed the highest resistance to cephalexin (96%, 95% CI: 80.46–99.80%), followed by ampicillin (76%, 95% CI: 56.57–88.50%), azithromycin (56%, 95% CI: 37.07–73.33%), tetracycline (44%, 95% CI: 26.67–62.93%), cotrimoxazole (40%, 95% CI: 23.40–59.26%), ciprofloxacin and levofloxacin (36%, 95% CI: 20.25–55.48%), gentamicin (24%, 95% CI: 11.50–43.43%), cefotaxime (20%, 95% CI: 8.86–39.13%), ceftriaxone and ceftazidime (12%, 95% CI: 4.17–29.96%), and fosfomycin (4%, 95% CI: 0.21–19.54%) (Figure 1). In addition, 100% of the isolates exhibited sensitivity to nitrofurantoin and chloramphenicol (Figure 1).

In bivariate analysis, we observed very high positive significant correlations between resistance patterns against cotrimoxazole and tetracycline (*p* < 0.001); cotrimoxazole and ciprofloxacin (*p* < 0.001); tetracycline and ciprofloxacin (*p* < 0.001); tetracycline and azithromycin (*p* < 0.001); ceftazidime and cefotaxime (*p* < 0.001); cotrimoxazole and azithromycin (*p* < 0.001); cotrimoxazole and gentamycin (*p* < 0.001); levofloxacin and tetracycline (*p* < 0.001); azithromycin and ciprofloxacin (*p* < 0.001); levofloxacin and ciprofloxacin (*p* < 0.001); tetracycline and gentamycin (*p* < 0.01); ceftazidime and ceftriaxone (*p* < 0.01); levofloxacin and cotrimoxazole (*p* < 0.01); and levofloxacin and gentamycin (*p* < 0.01) (Supplementary Table S1). We also found moderate-to-lower significant positive correlations between resistance patterns of *C. freundii* isolates against different antibiotics (Supplementary Table S1).

### 2.3. MDR and MAR Profiles of C. freundii

The majority of the *C. freundii* isolates (15/25, 60%, 95% CI: 40.74–76.60%) were phenotypically MDR and showed a multiple antibiotic resistance (MAR) index of more than 0.2. Fourteen antibiotic resistance patterns were observed, and eleven of them were MDR. The most common MDR pattern was no. 3 (gentamicin-ciprofloxacin-cephalexin-azithromycin-tetracycline-ampicillin-cotrimoxazole-levofloxacin), which was 26.67% (4/15) of the MDR *C. freundii* isolates. One isolate was resistant to 11 antibiotics (out of 14 tested antibiotics) from seven different classes (out of ten classes) (Table 2). Moreover, the MAR index of *C. freundii* isolates varied from 0.07 to 0.79 (Table 2).

### 2.4. Genotypic Resistance Profiles of C. freundii Isolates

**Figure 2:**
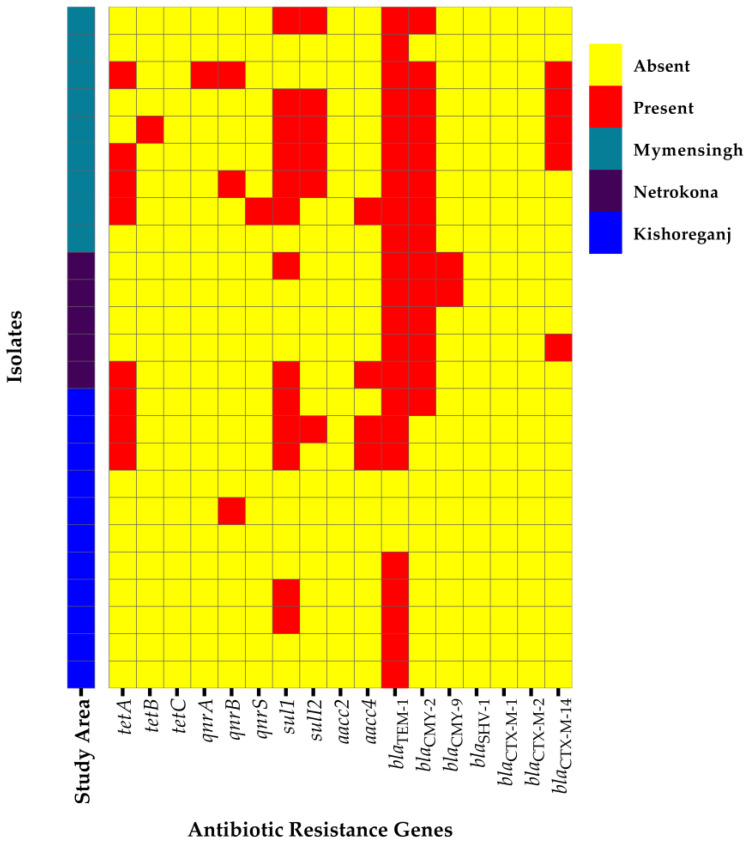
Heatmap showing the distribution of various antibiotic resistance genes of *C. freundii* isolated from cloacal swabs of ducks in Bangladesh.

Upon PCR analysis, out of 25 *C. freundii* isolates, beta-lactamase genes *bla*_TEM-1_, *bla*_CMY-2_, *bla*_CMY-9_, and *bla*_CTX-M-14_ were detected in 88% (95% CI: 70.04–95.83%), 56% (95% CI: 37.07–73.33%), 8% (95% CI: 1.42–24.97%), and 20% (95% CI: 8.86–39.13%) of the isolates, respectively (Figure 2). Genes conferring resistance to sulfonamides [*sul1*-52% (13/25), 95% CI: 33.49–69.97%; *sul2*-24% (6/25), 95% CI: 11.49–43.43%], tetracyclines [*tetA*-32% (8/25), 95% CI: 17.21–51.59%; *tetB*-4% (1/25), 95% CI: 0.21–19.54%], fluoroquinolones [*qnrA*-4% (1/25), 95% CI: 0.21–19.54%; *qnrB*-12% (3/25), 95% CI: 4.17–29.96%; *qnrS*-4% (1/25), 95% CI: 0.21–19.54%], and aminoglycosides [*aacC4*-16% (4/25), 95% CI: 6.40–34.65%] were also detected in the isolated *C. freundii*. No isolates harbored *bla*_SHV-1_, *bla*_CTX-M-1_, *bla*_CTX-M-2_, *tetC*, and *aacC2* genes (Figure 2).

In the bivariate analysis, a high positive significant correlation was observed between the presence of antibiotic resistance genes *aac4* and *tetA* (Pearson correlation coefficient, *ρ* = 0.636; *p* = 0.001), *qnrA* and *qnrB* (*ρ* = 0.553; *p* = 0.004), and *sul1* and *sul2* (*ρ* = 0.540; *p* = 0.005) (Supplementary Table S2). Moreover, moderate-to-low positive significant correlations were also observed between the presence of resistance genes *tetA* and *sul1* (*ρ* = 0.487; *p* = 0.013), *qnrS* and *aacC4* (*ρ* = 0.468; *p* = 0.018), *bla*_CMY-2_ and *bla*_CTX-M-14_ (*ρ* = 0.443; *p* = 0.026), *bla*_CTX-M-14_ and *sul2* (*ρ* = 0.421; *p* = 0.036), *aac4* and *sul1* (*ρ* = 0.419; *p* = 0.037), *bla*_TEM-1_ and *bla*_CMY-2_ (*ρ* = 0.417; *p* = 0.038), *tetB* and *bla*_CTX-M-14_ (*ρ* = 0.408; *p* = 0.043), and *qnrA* and *bla*_CTX-M-14_ (*ρ* = 0.408; *p* = 0.043) (Supplementary Table S2).

### 2.5. Comparison of Phenotypic and Genotypic Resistance Profiles of Isolated C. freundii

In bivariate analysis, a positive significant correlation was observed between phenotypic and genotypic resistance profiles of *C. freundii* isolates against tetracyclines (*ρ* = 0.846; *p* < 0.001), sulfonamides (*ρ* = 0.784; *p* < 0.001), aminoglycosides (*ρ* = 0.521; *p* = 0.008), and fluoroquinolones (*ρ* = 0.417; *p* = 0.038). Given that all the isolates were phenotypically resistant to at least one beta-lactam antibiotic (constant variable), we could not compute the Pearson correlation coefficient to show the correlation between phenotypic and genotypic resistant profiles of *C. freundii* isolates against beta-lactams. However, 88% similarity was exhibited between beta-lactam antibiotics (*n* = 25) and beta-lactamase genes (*n* = 22) based on the phenotypic (disk diffusion) and genotypic (PCR) assays.

## 3. Discussion

Ducks have the potential to harbor hazardous bacteria that can cause zoonotic diseases in humans, including salmonellosis, *E. coli* infections, cholera, psittacosis, and others [9]. In our study, about 16.67% of the duck samples harbored *C. freundii*. The detection of *C. freundii* in this study suggests that ducks have the potential to transfer this organism to humans, posing an important threat to public health. In Bangladesh, poultry farmers, surrounding people, and the environment are directly exposed to domestic ducks during the process of rearing them. As a result, people in direct contact with ducks, both on farms and in houses, are at an increased risk of being infected with *C. freundii* from ducks. Importantly, *C. freundii* isolates have the ability to cause bacteremia in humans, indicating a high risk to human health [4]. A previous study in South Korea reported that *C. freundii* bacteremia was the major risk factor for a higher mortality rate in hospitalized patients (aged ≥ 15 years) [26]. Duck droppings can contaminate the agricultural environment and can be a source of infections for crop farmers and other people exposed to the contaminated environment [27].

Over time, *C. freundii* has the tendency to develop resistance to different classes of broad-spectrum antibiotics, and a major emerging issue is the rapid spread of this antibiotic resistance [26]. The result of the antibiotic susceptibility test showed that *C. freundii* isolates showed resistance to different classes of broad-spectrum antibiotics. For example, resistance was found to multiple classes of antibiotics, including beta-lactams, sulfonamides, fluoroquinolones, tetracyclines, aminoglycosides, macrolides, and glycopeptides, and these antibiotics are used extensively in both human and veterinary medicine. Our study indicates that domestic ducks foraging in different environmental niches could be a potential carrier of antibiotic resistance. Similar to our study, Olaiton et al. [8] also showed that *C. freundii* isolated from duck droppings showed resistance to tetracyclines, aminoglycosides, beta-lactams, and sulfonamides.

Cephalosporins are still reliable antibiotics for the treatment of *C. freundii* infections, and the rapid emergence of cephalosporin resistance in *C. freundii* is considered a global health problem [4]. In this study, *C. freundii* isolates showed phenotypic resistance to ampicillin, cephalexin, cefotaxime, ceftriaxone, and ceftazidime. In addition to that, several genotypes associated with cephalosporin resistance were found. For example, *bla*_CMY-2_ (56%), *bla*_CMY-9_ (8%), and *bla*_CTX-M-14_ (20%) are considered clinically associated biomarkers [28]. The widespread distribution of beta-lactam genotypes in humans, animals, and the environment indicates an immediate need for improvement in the treatment of infectious diseases [29]. Resistance to beta-lactam antibiotics in an organism may also be developed due to the acquisition of other beta-lactamase genes, e.g., *bla*_TEM_ and *bla*_CMY_, in humans and animals [29,30]. The presence of these clinical biomarkers in *C. freundii* isolated from ducks is a clear indication of potential widescale spread from human *C. freundii* isolates or other members of the *Enterobacteriaceae* family.

Sulfonamides are a significant class of synthetic bacteriostatic antibiotics that are still widely used to treat bacterial infections in veterinary medicine [31]. In our study, 40% of the *C. freundii* isolates were resistant to the sulfonamide drug called cotrimoxazole. Resistance to sulfonamides in gram-negative bacteria, including *C. freundii*, typically results from the acquisition of one of two genes, *sul1* or *sul2*, which encode for dihydropteroate synthase forms that are not inhibited by the drug [32]. Sulfonamide resistance genes *sul1* and *sul2* were detected in 52% and 24% of the *C. freundii* isolates, respectively. One possible explanation for the increased occurrence of sulfonamide resistance genes among *C. freundii* isolates collected from the cloaca of ducks is the widespread abuse of this kind of antibiotic in the poultry industry. The presence of sulfonamide-resistant *C. freundii* and their corresponding genes in ducks should be concerning because these resistant bacteria have the potential to be transferred to humans via direct or indirect contact [33].

Resistance to fluoroquinolone antibiotics is an urgent problem in both human and veterinary medicine worldwide. More than 44% of *C. freundii* isolates showed phenotypic resistance to fluoroquinolone antibiotics, either to ciprofloxacin or to levofloxacin, which is an important public health concern as these drugs are widely used for humans and large animals. In general, the fluoroquinolone group of antibiotics is a reliable antimicrobial agent for the treatment of *Citrobacter* infections, especially *C. freundii* bacteremia [4]. In this study, we detected a higher rate of fluoroquinolone resistance gene *qnrB* (12%) than that of other *qnr* (*qnrA* and *qnrS*) genes, which is not unusual. The *qnrB* genes, which encode proteins liable for reduced susceptibility to fluoroquinolones, are by far the most common and diverse subfamily of *qnr* genes [34]. These antibiotic-resistant bacteria and their resistance genes have the potential to spread to humans via the food chain [35]. Given that these resistant isolates can be passed from ducks to humans, the use of fluoroquinolones in poultry needs to be closely regulated in order to avoid any further resistance to fluoroquinolones.

Tetracyclines are commonly used as one of the first-line antibiotics against a wide variety of non-life-threatening infections, including *C. freundii* infections [36]. The development of antibiotic resistance in *C. freundii* limits the treatment options. We report 44% of the *C. freundii* isolates were phenotypically resistant to tetracycline. We also detected tetracycline resistance genes *tetA* (8/25) and *tetB* (1/25) in the *C. freundii* isolates; however, none for the *tetC* gene. The detection of *tetA* and *tetB* genes in tetracycline-resistant *C. freundii* isolates demonstrates that the active efflux system was the initial mechanism of tetracycline resistance in ducks [37,38].

Aminoglycosides are among the most effective antibiotics available for treating serious infections [39]. In the present study, 24% of the *C. freundii* isolates were phenotypically resistant to the aminoglycoside antibiotic named gentamicin. Moreover, we detected the aminoglycoside resistance gene *aacC4* in 16% of the *C. freundii* isolates. The presence of this gene with significance to public health in ducks highlights the necessity of conducting an additional investigation into duck reservoirs for AMR. One of the important causal agents of community-acquired sepsis is *C. freundii* [40]. Therefore, the presence of aminoglycoside resistance in *C. freundii* may limit the treatment options for community-acquired sepsis because combining an aminoglycoside with a beta-lactam antibiotic and metronidazole is a common and effective experimental treatment for community-acquired sepsis [41].

This study found that the phenotypic and genotypic resistance profiles of *C. freundii* isolates against tetracyclines, sulfonamides, aminoglycosides, and fluoroquinolones were significantly correlated (*p* < 0.05). However, some isolates exhibited phenotypic susceptibility in the presence of resistance genes, and some isolates appeared phenotypic resistant but did not harbor resistant genes. Over time, several random mutations can occur in the gene sequence of an antibiotic resistance gene, rendering it inactive and transforming it into a resistance pseudogene that does not show the expected resistance characteristics [42]. Moreover, observed variations in susceptibility may be explained by heteroresistance processes such as random tandem gene amplification, uncommon mutation, and environmental manipulation of resistant genes [43]. The antibiotic may also act as a modulator, causing antibiotic-resistant genes to express poorly in vitro [44]. Therefore, whole-genome sequencing-based analyses have the potential to provide a precise genotype-to-phenotype resistance link.

Infections developed by MDR bacteria (with a high MAR index) have the potential to have severe repercussions for both human and animal health [45]. Humans and animals alike are at risk due to the spread of MDR *Citrobacter* spp. [46]. Infections caused by MDR *C. freundii* have fewer treatment options. In this study, 60% of the isolates were MDR, indicating an alarming issue in ducks and humans. The transfer of resistance genes from one resistant bacterium to another can lead to the development of MDR in bacteria that normally respond to the related classes of antibiotics [7]. Moreover, 60% of the isolates had a MAR index greater than 0.2, suggesting that these bacterial strains originated from a high-risk source of contamination in an area where antibiotics are frequently used [47]. Ducks can spread these pathogens to humans through the food chain or through direct contact, and they can also spread them to the environment through polluted water or feed [48,49].

## 4. Materials and Methods

### 4.1. Study Area

**Figure 3:**
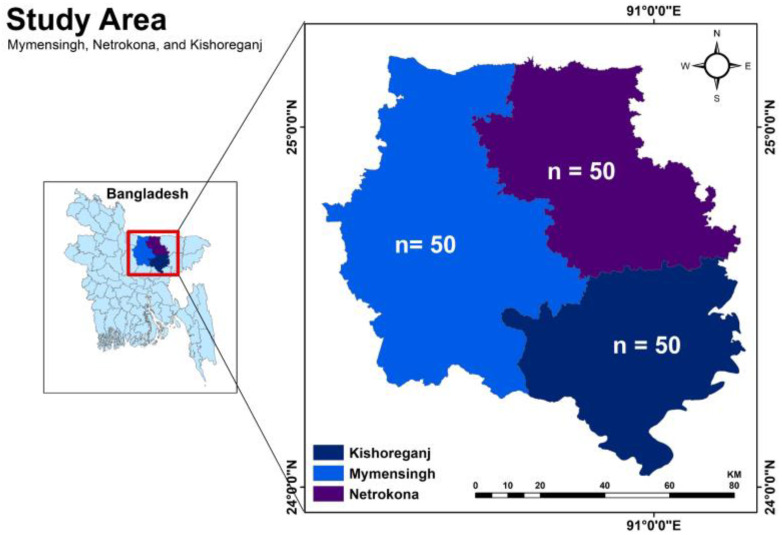
Maps showing study areas in Bangladesh with the number of samples collected from each district.

From January 2020 to January 2022, the present study was carried out in Mymensingh (24.7539° N, 90.4073° E), Netrokona (24.8103° N, 90.8656° E), and Kishoreganj (24.4260° N, 90.9821° E) districts of Bangladesh (Figure 3). The locations were chosen because of the high density of ducks in these districts, with massive wetland areas.

### 4.2. Sample Collection and Processing

We collected a total of 150 cloacal swabs of diseased domestic ducks (*Anas platyrhynchos domesticus*) (50 from each district) from different households (10 households from each district, each household reared domestic ducks with a range of 50–100). The diseased ducks had several symptoms, such as whitish or greenish diarrhea, leg weakness, headshaking, and even sudden death. Moreover, the ducks were reared in a scavenging or semi-scavenging system. The cloacal swab was transferred aseptically to a test tube containing Luria–Bertani (LB) broth (HiMedia, Maharashtra, India). The samples were transferred to the lab in a cold chain and enriched the bacteria by incubating the test tube contents of the samples aerobically for 18 to 24 h at 37 °C.

### 4.3. Isolation and Molecular Detection of Citrobacter *spp.*

One loopful (1–2 µL) of the overnight growth culture was streaked on a xylose-lysine deoxycholate (XLD) agar (HiMedia, India) plate, and the plate was then incubated aerobically for 24–48 h at 37 °C. For pure colonies, subcultures were done following the same procedures. Colonies showing yellow color with a black center on the XLD agar plate were assumed to be *Citrobacter* spp. isolates. Finally, we screened the single pure colonies for further confirmation by Gram’s staining technique and corresponding biochemical tests (such as urease, citrate, catalase, motility, H_2_S test, methyl red, Voges–Proskauer, and sugar fermentation tests) [50].

We performed a PCR test for the final confirmation of isolated *Citrobacter* spp. targeting a 16S rRNA gene (F: AGAGTTTGATCMTGGCTCAG, R: TACGGYTACCTTGTTACGACTT) as previously mentioned [51]. For PCR, the DNA from the bacterial genome was extracted by following the boiling and freeze–thawing methods described earlier [52,53]. Each extracted DNA was then amplified using a total of 20 µL of the final volume, including 10 µL of the master mix (2×) (Promega, Madison, WI, USA), 1 µL of each primer (forward and reverse) (100 pmol) (Macrogen, Republic of Korea), 2 µL of nuclease-free water, and 6 µL of genomic DNA. Amplified PCR products were examined on a 1.5% agarose gel (Invitrogen, Waltham, MA, USA) using a gel electrophoresis apparatus (Nippon Genetics, Tokyo, Japan). Amplicon products were observed under an ultraviolet trans-illuminator (Biometra, Göttingen, Germany) after being stained with ethidium bromide. The amplicon size was checked using a 1 kb DNA ladder (Promega, Madison, WI, USA).

### 4.4. Detection of C. freundii by MALDI-TOF Mass Spectrometry

*Citrobacter* spp. isolates that were positive in PCR assay were then subjected to matrix-assisted laser desorption/ionization time-of-flight (MALDI-TOF) mass spectrometry to detect *C. freundii* isolates. The MALDI-TOF analysis was done in the QC Laboratory, Dhaka, Bangladesh, and was followed by the procedures previously described by Kolínská et al. [54].

### 4.5. Antibiotic Susceptibility Test

#### 4.5.1. Phenotypic Analysis

According to the Clinical and Laboratory Standards Institute [55], the AST of isolated *C. freundii* was done by a Kirby–Bauer disk diffusion test [56]. In this study, 14 antibiotics from ten classes were chosen based on their availability in Bangladesh: fluoroquinolones (ciprofloxacin—5 μg, levofloxacin—5 μg), aminoglycosides (gentamicin—10 μg), tetracyclines (tetracycline—30 μg), macrolides (azithromycin—15 μg), cephalosporins (ceftriaxone—30 μg, cephalexin—30 μg, cefotaxime—30 μg, ceftazidime—30 μg), penicillins (ampicillin—10 μg), glycopeptides (chloramphenicol—30 μg), sulfonamides (cotrimoxazole- 25 μg), phosphonic acids (fosfomycin—200 μg), nitrofurantoin (nitrofurantoin—100 μg) (HiMedia, India). A multidrug-resistant (MDR) isolate was characterized as one that is resistant to three or more antibiotic classes [57]. We enumerated the multiple antibiotic resistance (MAR) index by the following formula [47]: MAR = *w*/*v*; here, w = number of antibiotics to which an isolate is resistant, v = total number of antibiotics used in this study.

#### 4.5.2. Genotypic Analysis

Genes conferring resistance to beta-lactams (*bla*_TEM-1_, *bla*_CMY-2_, *bla*_CMY-9_, *bla*_SHV-1_, *bla*_CTX-M-1_, *bla*_CTX-M-2_, and *bla*_CTX-M-14_), sulfonamides (*sul1* and *sul2*), tetracyclines (*tetA*, *tetB*, and *tetC*), fluoroquinolones (*qnrA*, *qnrB*, and *qnrS*), and aminoglycosides (*aacC2* and *aacC4*) were tested by a simplex PCR assay (Table 3).

### 4.6. Statistical Analyses

#### 4.6.1. Descriptive Analysis

We used Microsoft Excel 2013 (Los Angeles, CA, USA) for data entry and the Statistical Package for Social Science (SPSS 25, IBM, Chicago, IL, USA) and GraphPad Prism 8.4.2 (GraphPad Software, Inc., Avenida De La Playa La Jolla, CA, USA) for the data analysis. We performed the chi-square test for relatedness (with a *Z*-test for proportion) to understand the variations in the prevalence of *C. freundii* among sampling sites. Statistical significance was defined as a *p*-value of less than 0.05. The binomial 95% confidence interval (CI) was calculated following the Wilson and Brown Hybrid method [60]. Using GraphPad Prism, we created the heatmap to show the distribution of phenotypic and genotypic antibiotic resistance profiles of *C. freundii* isolates.

#### 4.6.2. Bivariate Analysis

We performed bivariate analysis in SPSS to determine whether resistance patterns in pairs of antibiotics and antibiotic resistance genes from isolated *C. freundii* were correlated. Moreover, the correlation between phenotypic and genotypic resistance profiles of *C. freundii* isolates against different classes of antibiotics was determined using bivariate analysis. A statistically significant *p*-value was less than 0.05 (*p* < 0.05).

## 5. Conclusions

*Citrobacter* spp. was found to be present in more than 16% of the samples collected from ducks in Bangladesh. We reported a range of 20% to 96% resistance in *C. freundii* isolates to different important antibiotics. The detection of genes encoding resistance to various classes of antibiotics in *C. freundii* isolated from ducks indicates a significant risk to human health due to the widespread presence of antibiotic resistance and their associated resistance genes in *C. freundii*. Given the close relationship between ducks, water, and other environmental components, there is a concern about the spreading of antibiotic-resistant *C. freundii* to humans. This may pose a potential human health risk. *C. freundii* should be characterized more elaborately using whole genome sequencing. However, further studies using the One Health approach and developed tools and high technologies are helpful in understanding the potential impact of MDR *C. freundii* in humans and animals and to minimize the risk of the emergence of MDR *C. freundii* in both animals and humans.

## Figures and Tables

**Table 1:** Prevalence of *C. freundii* isolated from cloacal swabs of ducks from different districts of Bangladesh.

Locations	No. of Samples Collected	No. of Positive Isolates (%)	95% CI (%)	*p*-Value
Mymensingh	50	9 (18 ^a^)	9.77–30.80	0.261
Netrokona	50	5 (10 ^a^)	4.35–21.36	
Kishoreganj	50	11 (22 ^a^)	12.75–35.24	
Overall	150	25 (16.67)	11.55–23.45	

Values with different superscripts differ significantly (*p* < 0.05) within the variable under assessment, CI = Confidence interval.

**Table 2:** Phenotypic multidrug resistance profiles and multiple antibiotic resistance index profiles of *C. freundii* isolated from cloacal swabs of ducks.

Pattern No.	Antibiotic Resistance Patterns	No. of Antibiotics (Classes)	No. of MDR Isolates (%)	MAR Index
1	CN, CIP, CL, AZM, TE, AMP, CRO, COT, CTX, CAZ, LEV	11 (7)	1 (6.67)	0.79
2	CIP, CL, AZM, TE, AMP, CRO, COT, CTX, CAZ, LEV	10 (6)	1 (6.67)	0.71
3	CN, CIP, CL, AZM, TE, AMP, COT, LEV	8 (7)	4 (26.67)	0.57
4	CN, CIP, CL, AZM, TE, AMP, COT, CTX	8 (7)	1 (6.67)	0.57
5	CIP, CL, AZM, TE, AMP, COT, LEV	7 (6)	1 (6.67)	0.50
6	CIP, CL, AZM, TE, AMP, COT	6 (6)	1 (6.67)	0.43
7	CL, AZM, TE, AMP, COT, CRO	6 (5)	1 (6.66)	0.43
8	CL, AMP, LEV, CTX, CAZ	5 (3)	1 (6.67)	0.36
9	CL, AZM, AMP, FO	4 (4)	1 (6.67)	0.29
10	CL, AZM, TE, LEV	4 (4)	1 (6.67)	0.29
11	CL, AZM, AMP	3 (3)	2 (13.33)	0.21
12 *	CL, AMP	2 (2)	4 *	0.14
13 *	CL	1 (1)	5 *	0.07
14 *	AMP	1 (1)	1 *	0.07

MDR = multidrug-resistant, MAR = multiple antibiotic resistance, LEV = Levofloxacin; CAZ = ceftazidime; CTX = cefotaxime; FO = fosfomycin; COT = cotrimoxazole; CRO = ceftriaxone; AMP = ampicillin; TE = tetracycline; AZM = azithromycin; CL = Cephalexin; CIP = ciprofloxacin; CN = gentamycin; * Non-multidrug-resistant.

**Table 3:** Primers used in the present study for detecting *Citrobacter* spp. and different antibiotic resistance genes in *C. freundii* isolates from cloacal swabs of ducks.

Factors	Target Genes	Primer Sequences (5′-3′)	Annealing Temp.	Amplicon Size (bp)	References
Beta-lactamase	*bla*_TEM-1_	F-CAGCGGTAAGATCCTTGAGA R-ACTCCCCGTCGTGTAGATAA	55	643	[58]
	*bla*_CMY-2_	F-TGGCCGTTGCCGTTATCTAC R-CCCGTTTTATGCACCCATGA	55	870	[58]
	*bla*_CMY-9_	F-TCAGCGAGCAGACCCTGTTC R-CTGGCCGGGATGGGATAGTT	55	874	[58]
	*bla*_SHV-1_	F-GGCCGCGTAGGCATGATAGA R-CCCGGCGATTTGCTGATTTC	55	714	[58]
	*bla*_CTXM-2_	F-GGCGTTGCGCTGATTAACAC R-TTGCCCTTAAGCCACGTCAC	55	486	[58]
	*bla*_CTX-M-1_	F-AACCGTCACGCTGTTGTTAG R-TTGAGGCTGGGTGAAGTAAG	55	766	[58]
	*bla*_CTX-M-14_	F-GCCTGCCGATCTGGTTAACT R-GCCGGTCGTATTGCCTTTGA	55	358	[58]
Tetracyclines	*tetA*	F-GCGCCTTTCCTTTGGGTTCT R-CCACCCGTTCCACGTTGTTA	55	831	[58]
	*tetB*	F-CCCAGTGCTGTTGTTGTCAT R-CCACCACCAGCCAATAAAAT	55	723	[58]
	*tetC*	F-TTGCGGGATATCGTCCATTC R-CATGCCAACCCGTTCCATGT	54	1019	[58]
Fluroquinolones	*qnrA*	F-TCAGCAAGAGGATTTCTCA R-GGCAGCACTATTACTCCCA	55	670	[59]
	*qnrB*	F-ATGACGCCATTACTGTATAA R-GATCGCAATGTGTGAAGTTT	53	680	[59]
	*qnrS*	F-ACGACATTCGTCAACTGCAA R-TAAATTGGCACCCTGTAGGC	54	428	[58]
Sulfonamides	*sul1*	F-TCACCGAGGACTCCTTCTTC R-CAGTCCGCCTCAGCAATATC	55	331	[58]
	*sul2*	F-CCTGTTTCGTCCGACACAGA R-GAAGCGCAGCCGCAATTCAT	55	435	[58]
Aminoglycosides	*aacC2*	F-GGCAATAACGGAGGCAATTCGA R-CTCGATGGCGACCGAGCTTCA	55	450	[58]
	*aacC4*	F-ACTGAGCATGACCTTGCGATGCTCTA R-TACCTTGCCTCTCAAACCCCGCTT	55	436	[58]

## Data Availability

The datasets used and/or analyzed during the current study are available from the corresponding author upon request.

## Supplementary Materials

The following supporting information can be downloaded at: https://www.mdpi.com/article/10.3390/antibiotics12040769/s1, Table S1: Pearson correlation coefficients assessing correlation between pairs of antibiotics to which *C. freundii* isolates showed resistance; Table S2: Pearson correlation coefficients assessing correlation between pairs of antibiotic resistance genes in *C. freundii* isolates from cloacal swabs of ducks.
