# Supplementary material for: Phenotypic and Genotypic Characteristics of Antimicrobial Resistance in Citrobacter freundii Isolated from Domestic Ducks (Anas platyrhynchos domesticus) in Bangladesh
Source: Antibiotics (Basel). 2023 Apr 17;12(4):769. doi: 10.3390/antibiotics12040769 (PMC10135275; doi:10.3390/antibiotics12040769)
Supplement: Supplementary file 1 [file antibiotics-12-00769-s001.zip › antibiotics-2318113-supplementary.pdf]

Phenotypic and genotypic characteristics of antimicrobial resistance in Citrobacter freundii isolated from domestic ducks (*Anas platyrhynchos domesticus*) in Bangladesh

Tarana Ahmed [1,2,*], Md. Saiful Islam [1], Najmul Haider [3,4], Linzy Elton [5], Badrul Hasan [6], Mohammad Nuruzzaman [7], Md. Tanvir Rahman [1], S.M. Lutful Kabir [1], Md. Shahidur Rahman Khan [1,*]

[1]Department of Microbiology and Hygiene, Bangladesh Agricultural University, Mymensingh 2202, Bangladesh; dvm41257@bau.edu.bd (M.S.I.); tanvirahman@bau.edu.bd (M.T.R.); lkabir79@bau.edu.bd (S.M.L.K.).

[2]Department of Livestock Services, Ministry of Fisheries & Livestock, Government of the Peoples Republic of Bangladesh, Krishi Khamar Sarak, Farmgate, Dhaka-1215, Bangladesh.

[3]School of Life Sciences, Keele University, Staffordshire, United Kingdom; n.haider@keele.ac.uk (N.H.).

[4]The Royal Veterinary College, University of London, Hertfordshire, United Kingdom.

[5]Centre for Clinical Microbiology, Department of Infection, Division of Infection and Immunity, Royal Free Campus, University College London, London, UK; Linzy.Elton@ucl.ac.uk (L.E.)

[6]Department of Jobs, Precincts and Regions, AgriBio, Centre for AgriBioscience, Bundoora, Victoria, 3083, Australia; badrul.hasan@agriculture.vic.gov.au (B.H.)

[7]Ministry of Public Administration, Dhaka- 1205, Bangladesh; mnzaman27@gmail.com (M.N.)

[*]**Correspondence:** tarana_ahmed07@yahoo.com (T.A.) and msrkhan001@yahoo.com (M.S.R.K.)

Supplementary Table S1: Pearson correlation coefficients assessing correlation between pairs of antibiotics to which *C. freundii* isolates showed resistance.

		C	CN	CIP	CL	AZM	TE	AMP	CRO	COT	FO	F	CTX	CAZ	LEV
**C**	ϱ	.a	.a												
	Sig. (2-tailed)		.												
**CN**	ϱ	.a	1												
	Sig. (2-tailed)	.	.												
**CIP**	ϱ	.a	**.749****	1											
	Sig. (2-tailed)	.	**0.000**	.											
**CL**	ϱ	.a	0.115	0.153	1										
	Sig. (2-tailed)	.	0.585	0.465	.										
**AZM**	ϱ	.a	**.498***	**.665****	0.23	1									
	Sig. (2-tailed)	.	**0.011**	**0.000**	0.268	.									
**TE**	ϱ	.a	**.634****	**.846****	0.181	**.786****	1								
	Sig. (2-tailed)	.	**0.001**	**0.000**	0.387	**0.000**	.								
**AMP**	ϱ	.a	0.316	**.421***	-0.115	**.445***	0.309	1							
	Sig. (2-tailed)	.	0.124	**0.036**	0.585	**0.026**	0.132	.							
**CRO**	ϱ	.a	0.081	0.236	0.075	0.327	**.417***	0.208	1						
	Sig. (2-tailed)	.	0.701	0.256	0.72	0.11	**0.038**	0.32	.						
**COT**	ϱ	.a	**.688****	**.919****	0.167	**.724****	**.921****	**.459***	**.452***	1					
	Sig. (2-tailed)	.	**0.000**	**0.000**	0.426	**0.000**	**0.000**	**0.021**	**0.023**	.					
**FO**	ϱ	.a	-0.115	-0.153	0.042	0.181	-0.181	0.115	-0.075	-0.167	1				
	Sig. (2-tailed)	.	0.585	0.465	0.843	0.387	0.387	0.585	0.72	0.426	.				
**F**	ϱ	.a	.a	.a	.a	.a	.a	.a	.a	.a	.a	.a			
	Sig. (2-tailed)	.	.	.	.	.	.	.	.	.	.	.			
**CTX**	ϱ	.a	0.187	0.25	0.102	0.04	0.161	0.281	**.431***	0.204	-0.102	.a	1		
	Sig. (2-tailed)	.	0.37	0.228	0.627	0.848	0.442	0.174	**0.032**	0.328	0.627	.	.		
**CAZ**	ϱ	.a	0.081	0.236	0.075	0.079	0.169	0.208	**.621****	0.201	-0.075	.a	**.739****	1	
	Sig. (2-tailed)	.	0.701	0.256	0.72	0.706	0.42	0.32	**0.001**	0.335	0.72	.	**0.000**	.	

**LEV**	ϱ	.a	**.554****	**.653****	0.153	**.497***	**.678****	0.226	0.236	**.578****	-0.153	.a	0.25	**.492***	1
	Sig. (2-tailed)	.	**0.004**	**0.000**	0.465	**0.012**	**0.000**	0.277	0.256	**0.002**	0.465	.	0.228	**0.012**	.

Here, ** Correlation is significant at the 0.01 level (2-tailed); * Correlation is significant at the 0.05 level (2-tailed); [a]Cannot be computed because at least one of the variables is constant; ϱ = Pearson correlation coefficient; LEV = levofloxacin; CAZ = ceftazidime; CTX = cefotaxime; F = nitrofurantoin; FO = fosfomycin; COT = cotrimoxazole; CRO = ceftriaxone; AMP = ampicillin; TE = tetracycline; AZM = azithromycin; CL = Cephalexin; CIP = ciprofloxacin; CN = gentamycin; C = chloramphenicol.

Supplementary Table S2. Pearson correlation coefficients assessing correlation between pairs of antibiotic resistance genes in *C. freundii* isolates from cloacal swabs of ducks.

		bla$_{TEM-1}$	*bla*$_{CMY-2}$	*bla*$_{CMY-9}$	*bla*$_{CTX-M-1}$	*bla*$_{CTX-M-2}$	*bla*$_{CTX-M-14}$	*bla*$_{SHV-1}$	*sul1*	*sul2*	*tetA*	*tetB*	*tetC*	*qnrA*	*qnrB*	*qnrS*	*aacc2*	*aacc4*
bla$_{TEM-1}$	ϱ	1																
	p																	
bla$_{CMY-2}$	ϱ	**.417***	1															
	p	**0.038**																
bla$_{CMY-9}$	ϱ	0.109	0.261	1														
	p	0.604	0.207															
bla$_{CTX-M-1}$	ϱ	.b	.b	.b	.b													
	p	.	.	.														
bla$_{CTX-M-2}$	ϱ	.b	.b	.b	.b	.b												
	p	.	.	.	.													
bla$_{CTX-M-14}$	ϱ	0.185	**.443***	-0.147	.b	.b	1											
	p	0.377	**0.026**	0.482	.	.												
bla$_{SHV-1}$	ϱ	.b	.b	.b	.b	.b	.b	.b										
	p	.	.	.	.	.	.											
sul1	ϱ	0.384	0.277	-0.012	.b	.b	0.08	.b	1									
	p	0.058	0.179	0.955	.	.	0.704	.										
sulI2	ϱ	0.208	0.309	-0.166	.b	.b	**.421***	.b	**.540****	1								
	p	0.32	0.132	0.429	.	.	**0.036**	.	**0.005**									
tetA	ϱ	0.253	0.263	-0.202	.b	.b	0.086	.b	**.487***	0.217	1							
	p	0.222	0.205	0.332	.	.	0.684	.	**0.013**	0.298								
tetB	ϱ	0.075	0.181	-0.06	.b	.b	**.408***	.b	0.196	0.363	-0.14	1						
	p	0.72	0.387	0.775	.	.	**0.043**	.	0.347	0.074	0.504							
tetC	ϱ	.b	.b	.b	.b	.b	.b	.b	.b	.b	.b	.b	.b					
	p	.	.	.	.	.	.	.	.	.	.	.						

qnrA	ϱ	0.075	0.181	-0.06	.b	.b	**.408***	.b	-0.212	-0.115	0.298	-0.042	.b	1				
	p	0.72	0.387	0.775	.	.	**0.043**	.	0.308	0.585	0.149	0.843	.					
qnrB	ϱ	-0.242	0.079	-0.109	.b	.b	0.123	.b	-0.138	0.081	0.274	-0.075	.b	**.553****	1			
	p	0.243	0.706	0.604	.	.	0.558	.	0.511	0.701	0.184	0.72	.	**0.004**				
qnrS	ϱ	0.075	0.181	-0.06	.b	.b	-0.102	.b	0.196	-0.115	0.298	-0.042	.b	-0.042	-0.075	1		
	p	0.72	0.387	0.775	.	.	0.627	.	0.347	0.585	0.149	0.843	.	0.843	0.72			
aacc2	ϱ	.b	.b	.b	.b	.b	.b	.b	.b	.b	.b	.b	.b	.b	.b	.b	.b	
	p	.	.	.	.	.	.	.	.	.	.	.	.	.	.	.		
aacc4	ϱ	0.161	-0.053	-0.129	.b	.b	-0.218	.b	**.419***	0.01	**.636****	-0.089	.b	-0.089	-0.161	**.468***	.b	1
	p	0.442	0.802	0.54	.	.	0.295	.	**0.037**	0.961	**0.001**	0.672	.	0.672	0.442	**0.018**	.	

Here, **Correlation is significant at the 0.01 level (2-tailed); *Correlation is significant at the 0.05 level (2-tailed); [.b]Cannot be computed because at least one of the variables is constant; ϱ = Pearson correlation coefficient; *p* = Significance (2-tailed)
